# Data integration methodology that couples novel bioreactor monitoring tools, automated sampling, and applied mathematics to redefine bioproduction processes

**DOI:** 10.1186/1753-6561-7-S6-P115

**Published:** 2013-12-04

**Authors:** Lisa J Graham, Jeffrey F Breit, Lynn A Davis, Corey C Dow-Hygeland, Brandon J Downey

**Affiliations:** 1 Bend Research Inc

## 

Cell physiology dynamically affects the nutrient requirements of a culture. It is critical to obtain data over appropriate time intervals to assess the impact of process conditions on the cell population. By optimizing bioreactor operation, feed strategies and media composition, we can limit the number of experiments to obtain the empirical data sets.

For this poster, we present an emerging process-development methodology that is based on applying novel and existing bioreactor monitoring technologies, coupled with applied mathematics, to bioreactor processes. This approach employs tools like dielectric spectroscopy, aseptic autosamplers, and cell-based bioreactor models. We will illustrate how information gained from these tools can be coupled through utilization of the proper data integration and applied mathematics techniques.

The knowledge gained using this improved process development methodology also supports a less-invasive monitoring and feedback system, and can be implemented using a customized bioreactor control code.

